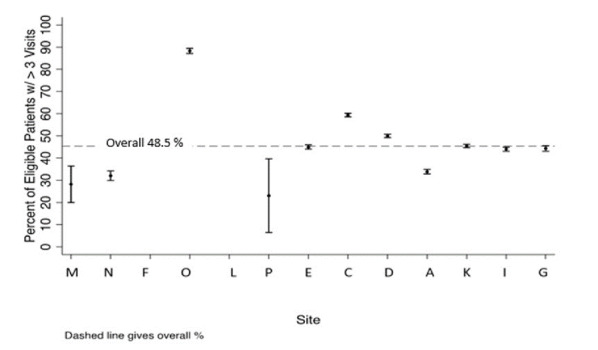# Testing an Automated Approach to Identify Variation in Outcomes among Children with Type 1 Diabetes across Multiple Sites

**DOI:** 10.1097/pq9.0000000000000602

**Published:** 2022-09-08

**Authors:** Jessica Addison, Hanieh Razzaghi, Charles Bailey, Kimberley Dickinson, Sarah D. Corathers, David M. Hartley, Levon Utidjian, Adam C. Carle, Erinn T. Rhodes, G. Todd Alonso, Michael J. Haller, Anthony W. Gannon, Justin A. Indyk, Ana Maria Arbeláez, Elizabeth Shenkman, Christopher B. Forrest, Daniel Eckrich, Brianna Magnusen, Sara Deakyne Davies, Kathleen E. Walsh

**Affiliations:** From the * Division of Adolescent and Young Adult Medicine, Boston Children’s Hospital, Boston, Mass.; † Applied Clinical Research Center, Children’s Hospital of Philadelphia, Philadelphia, Pa.; ‡ Department of Pediatrics, Perelman School of Medicine at the University of Pennsylvania, Pa.; § Division of Endocrinology, Cincinnati Children’s Hospital, Cincinnati, Ohio; ¶ Department of Pediatrics, University of Cincinnati College of Medicine, Cincinnati, Ohio; ∥ James M. Anderson Center for Health Systems Excellence, Cincinnati Children’s Hospital, Cincinnati, Ohio; ** Department of Psychology, College of Arts and Sciences, University of Cincinnati, Cincinnati, Ohio; †† Division of Endocrinology, Boston Children’s Hospital, Boston, Mass.; ‡‡ Department of Pediatrics, Harvard Medical School, Boston, Mass.; §§ University of Colorado Anschutz Medical Campus, Barbara Davis Center, Aurora, Colo.; ¶¶ Department of Pediatrics, University of Florida, Gainesville, Fla.; ∥∥ Nemours Children’s Hospital, Wilmington, Del.; *** Section of Endocrinology, Nationwide Children’s Hospital, Columbus, Ohio; ††† Department of Pediatrics, The Ohio State University College of Medicine, Columbus, Ohio; ‡‡‡ Washington University in St. Louis, St. Louis, Mo.; §§§ St. Louis Children’s Hospital, St. Louis, Mo.; ¶¶¶ University of Florida, College of Medicine, Department of Health Outcomes and Biomedical Informatics, Gainesville, Fla.; ∥∥∥ Division of General Pediatrics, Boston Children’s Hospital, Boston, Mass.

## Abstract

**Introduction:**

Efficient methods to obtain and benchmark national data are needed to improve comparative quality assessment for children with type 1 diabetes (T1D). PCORnet is a network of clinical data research networks whose infrastructure includes standardization to a Common Data Model (CDM) incorporating electronic health record (EHR)-derived data across multiple clinical institutions. The study aimed to determine the feasibility of the automated use of EHR data to assess comparative quality for T1D.

**Methods:**

In two PCORnet networks, PEDSnet and OneFlorida, the study assessed measures of glycemic control, diabetic ketoacidosis admissions, and clinic visits in 2016–2018 among youth 0–20 years of age. The study team developed measure EHR-based specifications, identified institution-specific rates using data stored in the CDM, and assessed agreement with manual chart review.

**Results:**

Among 9,740 youth with T1D across 12 institutions, one quarter (26%) had two or more measures of A1c greater than 9% annually (min 5%, max 47%). The median A1c was 8.5% (min site 7.9, max site 10.2). Overall, 4% were hospitalized for diabetic ketoacidosis (min 2%, max 8%). The predictive value of the PCORnet CDM was *>*75% for all measures and >90% for three measures.

**Conclusions:**

Using EHR-derived data to assess comparative quality for T1D is a valid, efficient, and reliable data collection tool for measuring T1D care and outcomes. Wide variations across institutions were observed, and even the best-performing institutions often failed to achieve the American Diabetes Association HbA1C goals (<7.5%).

## INTRODUCTION

Fewer than half of the 165,000 US youth under age 20 with Type 1 Diabetes (T1D) have optimal glycemic control, and approximately 33,000 (20%) have poor glycemic control, as measured by hemoglobin A1c (HbA1c).^1^ In one study, one in four teenagers with T1D made dangerous insulin dosing errors.^2^ The leading cause of death before age 30 among individuals with childhood-onset T1D is acute complications (eg, severe hypoglycemia, diabetic ketoacidosis).^3,4^ These findings highlight a need to improve outcomes nationally for children with T1D.

There has been increasing interest in quality of care and health outcomes among youth with T1D. Understanding variation in the quality of care and outcomes between health systems is an important early step in improving outcomes.^5,6^ Benchmarking measures of care quality, such as healthcare visits and glycemic control in this population, is a first step to improving care.

Existing healthcare quality measures among children with T1D include *US News and World Report* (*USNWR*) measures and T1Dx-QI group QI Collaborative.^7^ *USNWR* healthcare quality measures are used to inform their ratings for pediatric endocrine care, including youth up to age 18 years. T1D Exchange (https://t1dexchange.org/) is a nonprofit network of health systems that work together to evaluate care, provide education, and perform research to improve health outcomes in individuals with diabetes.^7^ T1D Exchange’s glycemic control and quality of care measures were very similar to those used by *USNWR*, but T1D Exchange follows patients across their lifespans.

Traditionally, health system quality measurement and benchmarking have employed billing data or manual chart review informed by prospective clinical registries; several groups have started to draw quality measure data directly from health centers’ electronic health record (EHR) data in an automated manner.^8–10^ In other words, a single electronic query is run on one or more health system’s clinical databases, using code such as SAS or R, producing a comparative quality of care report. One clinical data network available in the United States is PCORnet,^11^ a network of clinical research networks whose infrastructure improves interoperability among EHR-derived data from multiple health systems through rapid, automated data extraction and shared data governance. The PCORnet Common Data Model (CDM) provides standard definitions for data drawn from various clinical information systems into a common format (https://pcornet.org/data-driven-common-model/), which facilitates analyses across health systems. The study aimed to determine the feasibility of using EHR data to assess comparative quality for T1D.

## METHODS

This multisite retrospective cohort study used EHR data from 2016 to 2018 from hospital systems participating in two PCORnet Clinical Research Networks, PEDSnet (pedsnet.org) and OneFlorida (https://onefloridaconsortium.org/). We developed measure specifications, technical specifications, and final automated data extraction using the PCORnet CDM. In addition, we used chart review to assess the predictive value of quality measures. Cincinnati Children’s Hospital Medical Center served as the single Institutional Review Board of record for each network. Both networks had pre-existing internal data use agreements.

### Setting and Population

This study included all institutions in two PCORnet CRNs: PEDSnet and OneFlorida. PEDSnet (https://pedsnet.org) is a multispecialty network that conducts observational research and clinical trials across multiple children’s hospital health systems. At the time of this study, PEDSnet included eight children’s hospitals (**See Appendix A, Supplemental Digital Content 1,** which describes PEDSnet hospitals at the time of the study, http://links.lww.com/PQ9/A410). OneFlorida is a statewide clinical research network with 11 health care systems and affiliated practices serving all 67 counties in Florida (https://www.ctsi.ufl.edu/ctsa-consortium-projects/oneflorida/). OneFlorida includes two freestanding children’s hospitals and several institutions with pediatric care located within the larger health system. In 2016, these two data research networks included six million pediatric patients.

For each study site, we included all individuals younger than 21 years old who had at least one diagnosis term for T1D a year for more than 1 year and two or more T1D-related visits at the study site in the past year. We excluded youth with cystic fibrosis-related diabetes, steroid-induced diabetes, type 2 diabetes, gestational diabetes, and maturity-onset diabetes of the young.

### Measures

To select the focus measures for this study, a group of four parents of children with T1D and seven endocrinologists from PEDSnet and OneFlorida sites used the nominal group technique, a process for expert panel solution generation and decision-making.^12^ This time- and cost-efficient method facilitates equal participation from group members from different backgrounds, tolerates conflicting opinions, and mitigates facilitator bias.^12–14^ Over 2 weeks, this group generated and discussed ideas and voted for measures in teleconference meetings. In addition, we asked participants to select existing measures already in use at most or all institutions.

Table 1 shows specific measures used in this study with measure specifications in (**See Appendix B, Supplemental Digital Content 2,** which describes measure specifications, http://links.lww.com/PQ9/A411). The minimum eligibility criteria for inclusion were two or more visits per year. To assess glycemic control, we used the patient’s most recent A1c; for the assessment of 0.5% A1c improvement, we compared the two most recent A1c results.

**Table 1.** Measures of Type 1 Diabetes Care Quality Selected by Parents and Endocrinologists as the Focus for This Study and Whether Used by USNWR or T1D Exchange (for specifications, **see Appendix A, Supplemental Digital Content 1,** which describes PEDSnet hospitals at the time of the study, http://links.lww.com/PQ9/A410)

Measure (Assess over the Past 365 d)	Where Used
% patients with two or more measures of Hemoglobin A1c > 9%	T1D exchange
% patients with four or more visits to clinic for T1D in a year	USNWR
Median Hemoglobin A1c for clinic’s T1D patients	USNWR
% patients hospitalized for diabetic ketoacidosis in the past year	T1D Exchange
% patients with improvement in Hemoglobin A1c	T1D Exchange

#### Specification of Quality Measures

Using the PCORnet CDM, we mapped patient-level electronic data drawn directly from the EHRs^8^ sites to a common data dictionary (eg, same variable name, definition). The PCORnet CDM uses standard clinical coding systems, including International Statistical Classification of Diseases and Related Problems (ICD) 9 and 10, Current Procedural Terminology (CPT), Healthcare Common Procedure Coding System (HCPCS), Systematized Nomenclature of Medicine (SNOMED), and Logical Observation Identifiers Names and Codes (LOINC). The Data Coordinating Center at PEDSnet developed SAS code for each measure and then shared it with the Data Coordinating Center at OneFlorida. Both networks ran the code on CDM data from all sites. The Data Coordinating Center at OneFlorida then shared aggregated results back to PEDSnet’s Data Coordinating Center, which combined results from both networks. Finally, an endocrinologist and three health system analysts with expertise in reporting *USNWR* and T1D Exchange measures advised the research team to translate the quality-of-care criteria into measures that could be detected using the PCORnet CDM.

To identify diabetic ketoacidosis, we used identified SNOMED, ICD-9-CM, or ICD-10-CM codes for diabetic ketoacidosis associated with T1D in the EHR-derived data. We developed this approach informed by prior research that used ICD-9-CM and -10 codes in a Medicaid claims database that demonstrated a high positive predictive value (PPV) of diagnosis codes.^15^ Some patients might be admitted or transferred to study institutions from community hospitals at different stages of treatment and thus may have had resolution of acidosis or transitioned to subcutaneous insulin at the time of transfer. Using an algorithm based on laboratory data would miss these youth with DKA because the blood tests drawn at the receiving health system would not meet the criteria for DKA. For this reason, we used an algorithm that relies on SNOMED, ICD-9-CM, or ICD-10-CM codes.

### Chart Review

The eight largest pediatric sites were selected a priori to participate in a manual chart review to assess the predictive value of the CDM-derived measures (seven PEDSnet sites and one OneFlorida site). We determined the number of charts for each institution reviewed based on the size of their data contribution and staff availability. We randomly assigned an equal number of charts for youth who were within the criteria of the measures (eg, *>*4 clinic visits) and who were outside the criteria of the measures (eg, <4 clinic visits).

We developed and pilot-tested a chart review form (**See Appendix C, Supplemental Digital Content 3**, which describes chart review data collection form, http://links.lww.com/PQ9/A412). A chart reviewer, masked to CDM-derived measure results, reviewed the chart to determine whether the patient met the criteria to be included in the study and then reviewed the chart for each measure. The reviewer then opened the file with the CDM measure result for that patient and re-reviewed the chart to answer open-ended questions about reasons for any differences between the manual chart review and the CDM-derived measure results. We used REDCap Version 4.0 for chart review data collection.^16^ We trained chart reviewers at each site using a didactic webinar, as in prior research.^17^ To ensure consistency between institutions, we shared answers to questions raised during the chart review with all reviewers at each site.

### Analysis

We employed descriptive statistics to report rates for each measure overall and each institution. To calculate the PPV, we divided the number of youth where *both* the CDM-derived measure and chart indicated that the outcome occurred (eg, hospitalization for DKA) by the number of youth the CDM-derived measure indicated the outcome occurred. To calculate the negative predictive value (NPV), we divided the number of youth where the CDM-derived measure and chart indicated that the outcome did not occur (eg, not hospitalized for DKA) by the number of youth the CDM-derived measure indicated the outcome did not occur.

## RESULTS

There were 12 health systems with patients meeting inclusion criteria for the study, with 9,740 youth with T1D included; 10% percent were Black, and 13% were Hispanic. The average age was 15 years (interquartile range 6).

**Fig. 1.**
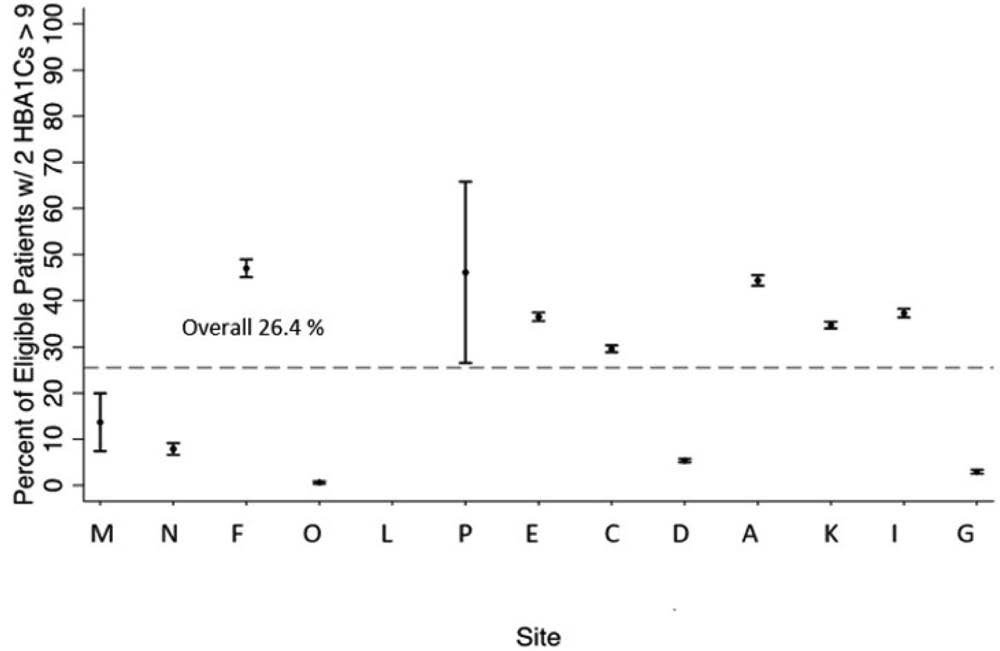
Percentage of youth with two or more measures of A1c > 9% and 95% confidence interval bars for each site.

Twenty-six percent of youth had two or more measures of A1c greater than 9% in the past year (Table 2). There was variation in both the success in meeting this measure and the predictive value of the data from each site (Fig. 1). Site D had excellent success on the measure (5.3%), and excellent PPVs (100%) for CDM-derived measures. Site G had excellent success (2.9%) with this measure but a lower PPV [71% (95% confidence interval [CI]: 53%–85%)]. In a review of 513 charts across the sites, if the CDM-derived measure specified that the child had had two or more measures of A1C greater than 9%, the chart agreed that 96% of the time (95% CI: 93%–97%; by site: 71%–100%). If the child did not have two or more measures of A1c over 9%, the chart agreed 100% of the time at all sites.

**Table 2.** Values for Hemoglobin A1c-related Measures Derived from the PCORnet Common Data Model, Ordered by Performance, and Predictive Value of Estimates Compared to Manual Chart Review

Measure	Site	No. Eligible Youth	Result (%)	No. Charts Reviewed	PPV^*^ (95% CI)	NPV^†^ (95% CI)
2 or more measures of hemoglobin A1c > 9 in prior year	G	646	2.9	35	71% (53%–85%)	100%
	D	1,769	5.3	60	100%	100%
	O	335	5.9	N/A	N/A	N/A
	N	200	7.9	N/A	N/A	N/A
	M	117	26.4	N/A	N/A	N/A
	C	1,554	29.5	58	95% (83%–99%)	100%
	K	1,776	34.7	58	95% (83%–99%)	100%
	E	1,171	36.5	60	99% (83%–99%)	100%
	I	1,165	37.3	60	97% (85%–99%)	100%
	A	824	44.3	60	100%	100%
	P	26	46.1	N/A	N/A	N/A
	F	289	47.0	120	99% (92%–99%)	100%
	Overall	9,740	26.4	513	96% (93%–97%)	100%
0.5% improvement in hemoglobin A1c	A	820	26.7	60	100%	100%
	F	282	26.0	120	100%	99% (92%–99%)
	E	1,168	25.1	60	83% (59%–96%)	100%
	I	1,152	24.6	60	100%	100%
	K	1,771	23.8	58	100%	100%
	C	1,530	23.8	58	82% (63%–94%)	100%
	O	9	14.6	N/A	N/A	N/A
	D	431	12.7	60	95% (76%–99%)	100%
	P	2	11.1	N/A	N/A	N/A
	N	88	8.6	N/A	N/A	N/A
	M	6	7.8	N/A	N/A	N/A
	G	641	1.1	35	13% (1%–53%)	46% (27%–67%)
	Overall	7,901	21.9	513	79% (76%–83%)	75% (71%–79%)

^*^PPV: the number of youth where both the Common Data Model (CDM) derived measure and chart indicated that the outcome occurred (eg, improvement in A1c) divided by number of youth where the measure derived from the CDM indicated the outcome occurred.

^†^ NPV: the number of youth where both the electronic measure and chart indicated that the outcome did not occur (eg, not hospitalized for DKA) divided by number of youth where the measure derived from the CDM indicated the outcome did not occur.

N/A, not applicable meaning this site was not a site identified at study inception and compensated to perform chart review.

Of study participants, 22% had an improvement of 0.5% or better in A1c in the past year. If the CDM-derived measure indicated that the child had a 0.5% improvement in their A1C, the chart agreed 79% of the time (95% CI: 76%–83%; by site: 13%–100%). If the CDM-derived measure did not indicate that the child had a 0.5% improvement in their A1c, the chart agreed 75% of the time (95% CI: 71%–79%; by site: 46%–100%). Failures in this CDM-derived measure were principally due to A1c laboratory results in the EHR from external databases (eg, outside labs) but not accessible to the CDM as discrete data points. Among institutions with good predictive value for the CDM-derived measure (*>*95% PPV and NPV) compared to chart review, improvement of *>*0.5% HbA1c varied from 13% to 27% (**See Appendix D, Supplemental Digital Content 4**, which describes figure of percentage of T1D youth with 0.5% or better improvement in their A1c for each site, with 95% CI bars, http://links.lww.com/PQ9/A413).

The median most recent A1c was 8.5% across the entire population (Table 3). Compared to the manual chart review, the predictive value of the CDM-derived measure of the most recent A1C was good; 94% (95% CI 92%–96%) of the time, the most recent A1c identified by the chart reviewer was the same value identified by the measure from the CDM. The most successful site (site N) had a median A1c of 7.9%. Four sites (sites M, G, P, and D) had higher median A1cs than average, ranging from 9.1% to 10.2% (**See Appendix E, Supplemental Digital Content 5,** which describes figure of the median of each patient’s most recent A1c with 95% CI bars for each site, http://links.lww.com/PQ9/A414).

**Table 3.** Values for Median A1c, Ordered by Result, Derived from the PCORnet Common Data Model and Percent Agreement where the Most Recent A1c Is the Same in the Common Data Model-derived Measure as in the Chart

Measure	Site	No. Eligible Youth	Result	No. Charts Reviewed	Percent Agreement (95% CI)
Median last hemoglobin A1c	N	200	7.9	N/A	N/A
	O	334	8.1	N/A	N/A
	C	1,554	8.3	58	100%
	K	1,776	8.4	58	100%
	E	1,171	8.5	60	98% (91%–100%)
	I	1,156	8.5	60	97% (84%–99%)
	A	824	8.8	60	100%
	F	290	9.0	120	87% (79%–93%)
	D	1,769	9.1	60	98% (91%–100%)
	P	3	9.6	N/A	N/A
	G	5,817	9.8	35	78% (62%–89%)
	M	13	10.2	N/A	N/A
	Overall	9,740	8.5	513	94% (92%–96%)

N/A, not applicable meaning this site was not a site identified at study inception and compensated to perform chart review.

During the study period, 4.2% of youth had hospital admissions for DKA by site: 2%–32%, (Table 4). Two smaller sites performed less than average on this measure (**See Appendix F, Supplemental Digital Content 6**, which describes percentage of T1D patients hospitalized for diabetic ketoacidosis in the past year (excluding patients diagnosed in the past year) for each site with 95% CI bars, http://links.lww.com/PQ9/A415). If the CDM-derived measure indicated that the child was hospitalized for DKA in the past year, the chart agreed 99% of the time (95% CI: 98%–100%; by site: 95%–100%). If it indicated that the child was not hospitalized for diabetic ketoacidosis, the chart agreed 91% of the time (95% CI: 87%–94%; by site: 37%–100%). One source of error for this measure was that the CDM identified a hospitalization as being for DKA when the chart review indicated the hospitalization was primarily for another reason (eg, surgery).

**Table 4.** Values for Utilization-related Measures Derived from the PCORnet Common Data Model, Ordered by Performance, and Predictive Value of Estimates Compared to Manual Chart Review

Measure	Site	No. Eligible Youth	Result (%)	No. Charts Reviewed	PPV^*^ (95% CI)	NPV^†^ (95% CI)
DKA hospital admissionsannually	O	334	1.6	N/A	N/A	N/A
	K	1,776	2.0	40	95% (74%–99%)	90% (68%–99%)
	P	3	4.2	N/A	N/A	N/A
	C	1,554	4.9	40	100%	100%
	I	1,156	4.9	40	100%	100%
	E	1,171	5.9	40	100%	100%
	F	290	5.9	40	100%	95% (75%–99%)
	D	1,769	7.1	40	100%	37% (19%–56%)
	G	5,817	7.2	40	100%	100%
	N	200	7.7	N/A	N/A	N/A
	A	824	8.3	41	100%	100%
	M	13	31.6	N/A	N/A	N/A
	Overall	9,740	4.2	320	99% (98%–100%)	91% (87%–94%)
4 or more T1D visits annually	O	334	88.3	N/A	N/A	N/A
	C	1,554	59.4	58	90% (74%–98%)	100%
	F	289	50.2	120	94% (85%–98%)	100%
	E	1,171	50.0	60	69% (48%–86%)	100%
	D	1,770	50.0	60	90% (74%–98%)	100%
	K	1,176	45.4	58	81% (63%–93%)	100%
	G	646	44.4	35	100%	64% (35%–87%)
	I	1,156	44.0	60	80% (61%–99%)	96% (81%–99%)
	A	824	33.9	60	92 (75%–99%)	85% (69%–95%)
	N	200	32.1	N/A	N/A	N/A
	M	13	28.2	N/A	N/A	N/A
	P	3	23.0	N/A	N/A	N/A
	Overall	9,740	48.5	513	78% (75%–82%)	76% (72%–79%)

^*^PPV: the number of youth where both the electronic measure and chart indicated that the outcome occurred (eg, hospitalization for DKA) divided by the number of youth where the measure derived from the CDM indicated the outcome occurred.

^†^NPV: the number of youth where both the electronic measure and chart indicated that the outcome did not occur (eg, not hospitalized for DKA) divided by the number of youth where the measure derived from the CDM indicated the outcome did not occur.

N/A, not applicable meaning this site was not a site identified at study inception and compensated to perform chart review.

Concerning clinic attendance, 48% of youth had four or more visits for T1D in the past year, from 23% to 88% by the site (**See Appendix G, Supplemental Digital Content 7**, which describes percentage of patients with four or more outpatient T1D visits in the past year for each site with 95% CI bars, http://links.lww.com/PQ9/A416). Compared to the chart review, the PPV for this CDM-derived measure was 78% (95% CI: 75%–82%), and the NPV was 76% (95% CI: 72%–79%) (Table 4). However, the CDM-derived measure sometimes incorrectly identified visits as related to T1D when they were not (eg, primary care visits) or missed T1D-related visits due to variation in visit labels between systems.

## DISCUSSION

This study investigates the utility of multi-center network research and its CDM’s potential to provide a more comprehensive perspective on the quality of care delivery in multiple clinical centers. Among the 12 institutions caring for children with T1D from two PCORnet networks, there was substantial variation in the quality of care and outcomes. Even the best sites in this study underperformed top-performing European centers, indicating an ongoing need for collaborative quality improvement.^18–20^ Data on variation in outcomes take a first step toward identifying best practices among top-performing US institutions for collaborative quality improvement initiatives. Furthermore, quality improvement collaboratives may leverage the research networks or the CDM approach to replace manual data collection and reporting for improved interoperability and efficiency.

Using data extracted automatically from the EHR, rather than reported by the 12 sites, overall rates of glycemic control and hospitalization for DKA in this study were consistent with prior US studies.^21–23^ Consistent with a few prior multinational studies that examined site-to-site differences, this study identified significant variation in utilization, glycemic control, and rates of DKA between sites.^18–20^ In all three multinational prior studies, several institutions and/or countries had a mean A1c (7.6% in all three studies) below the best-performing sites in our study. Some of the best clinics performed poorly in England, Wales, and the United States compared to top-performing countries. In England and Wales, as in the United States, there was a need to address poor performers and improve the performance of top-performing sites.

How does examining site-to-site variation in outcomes inform collaborative efforts to improve care for all children? Quality improvement collaboratives and researchers can use such data to identify best practices, examine the impact of different interventions for certain types of institutions, and provide a more accurate picture of the quality of care. High-performing sites can provide information on best practices for the rest of the sites.^5^ In fact, this type of collaborative benchmarking and sharing of lessons has effectively improved performance across quality improvement collaboratives.^24–26^ In addition, research on the impact of interventions on the quality of care in different institutions can help improve the intervention. For example, in response to failed implementation at some sites, the MARQUIS medication reconciliation study group made improvements to the intervention resulting in improved outcomes in the next implementation to a larger cohort.^27–29^ Finally, site-to-site variation can be an important indicator of overall quality. In one large European study, Charalampopoulos et al found significant variation between and within countries’ centers, concluding that average A1c levels are not an adequate summary of performance across a country or network.^19^ Our findings are consistent with their assertions. Overall rates pooled across the institutions were not an adequate summary of the state of T1D care at the 12 sites studied for any of the measures assessed due to the variation between institutions.

Institutional collaboratives focusing on quality improvement, such as T1D Exchange, are well-positioned to improve care across the United States for children with T1D. Using data from this study, one can identify best practices that can spread to other institutions. Such an approach has been highly successful for other quality improvement collaboratives such as the Solutions for Patient Safety Network.^25,30^ By collaborating to improve quality, low-performing health systems can benefit from successful strategies employed elsewhere. More importantly, even high-performing health systems can adopt best practices identified at other institutions or countries, moving the US median A1c closer to the goal of <7.0% set by the American Diabetes Association and International Society for Pediatric and Adolescent Diabetes.^31^

For collaborative quality improvement, rather than having institutions report data manually, obtaining data in an automated fashion directly from institutions’ EHRs using a common data dictionary, such as the PCORnet CDM, may help facilitate efficient data sharing and benchmarking.^8^ For three of the measures assessed in this study (hospitalization for DKA, two A1c > 9, and median last A1c), predictive values were above 90%. For the other two measures (>4 clinic visits for T1D and a 0.5% improvement in A1c), predictive values were above 70%. Ongoing opportunities for optimization of this tool include improved availability of data extracted into the EHR from external databases (eg, outside labs) and improved data structure at some institutions to enable this type of investigation. In addition, future studies could test the use of the CDM, or other similar automated approaches, with other ADA measures, such as the percent of patients with A1c < 7.5%.

According to one qualitative study, using CDMs for benchmarking is very important to pediatric health system leaders, and parents consistently expressed their need for health care quality data.^6^ Physician practices in the United States spend $1.5 billion annually (785 hours per physician) gathering and reporting quality of care metrics, but less than 30% use reported data to inform quality improvement.^32^ To achieve the best possible outcomes for youth and families, measures need to be more broad, fluid, and electronic.^33^

Although this is a large-scale study testing the use of data drawn from the EHR for benchmarking, it is important to note several limitations. First, we restricted the study population to pediatric patients from PEDSnet and OneFlorida. The results may not generalize to children with T1D cared for in general hospitals or community centers. Second, some OneFlorida sites had too few youths with T1D to measure healthcare quality reliably. We chose to include these sites rather than exclude smaller sites, to provide a picture of the range of potential variation in T1D care for children nationally. Although data elements needed for benchmarking may be similar, charting and coding practices may differ, altering data element behavior in the PCORnet CDM across sites. In particular, PEDSnet has noted that extensive standardization of ETL practices and the use of a CDM is an important contributor to semantic interoperability. Third, we conducted a chart review to assess the accuracy of electronic measures. Although the chart is a typical source of information about the reliability of health data in research, it is not a perfect record of a patient’s health. We did not explore patient-level predictors of glycemic control and outcomes published elsewhere,^4,34–37^ because the study intended to assess the value of measuring the outcomes using the PCORnet CDM for quality assessment and benchmarking. For the same reasons, we used existing measures rather than developing new measures. There were some limitations to these measures. For example, the measure of two A1cs *>* 9 excludes patients with only one A1c measured in the past year; and the PPV for 0.5% reduction in A1c was low (79%), potentially attributable to missing data or challenges with data capture. Last, although diabetes management requires a multidisciplinary team, including nurses, social workers, and nutritionists, we gathered input from clinicians, parents, and health system leaders.

This study had several strengths. First, it was a large observational study, including over 9,000 youth with T1D from across the nation, including large children’s hospitals and smaller care institutions. Second, the clinical research network infrastructure provides standardized, rapid, automated data extraction from the EHR and standardized data governance.^38^ Standardization allowed for interoperability and quicker identification of bugs and site-specific differences. Third, the incremental cost of repeating and refining measures was low, which allowed for iterative improvements during the study, making reassessment much more feasible over time. Finally, this study provided new information about the efficiency and validity of the network data query approach and variability in the quality of care metrics between institutions nationally.

### Conclusion Summary

This study found the use of PCORnet clinical research networks valid and efficient for extracting T1D quality of care measures from multiple health systems. In addition, the study identified variation in quality and outcomes, which may provide information for benchmarking and sharing best practices or future research. The long-term goal is to improve the efficiency, effectiveness, and utility of measuring and reporting health care quality for health systems and patients. This approach may have value for health systems, endocrinologists, patients, and families seeking to improve outcomes for youth with T1D.

## ACKNOWLEDGMENTS

We would like to acknowledge TX EQRO Medical Record Team for their thorough and thoughtful feedback on the chart review forms and methods. We would also like to thank Shannon Alford, MPH, CCRP, F. Sessions Cole, MD, Jonathan Finkelstein, MD, MPH, Susan Hague, MS, Rita Mangione-Smith, MD, MPH, Peter Margolis, MD, PhD, Jennifer McCafferty-Fernandez, PhD, Steve Muething, MD, Kathryn Ness, MD, MSCI, and Kathryn Obrynba, MD, for their support of the study.

## DISCLOSURE

K.E.W., within the past 36 months, has served as a consultant for Sanofi and Research Triangle Institute. The other authors have no financial interest to declare in relation to the content of this article.

## Supplementary Material



**Figure:**
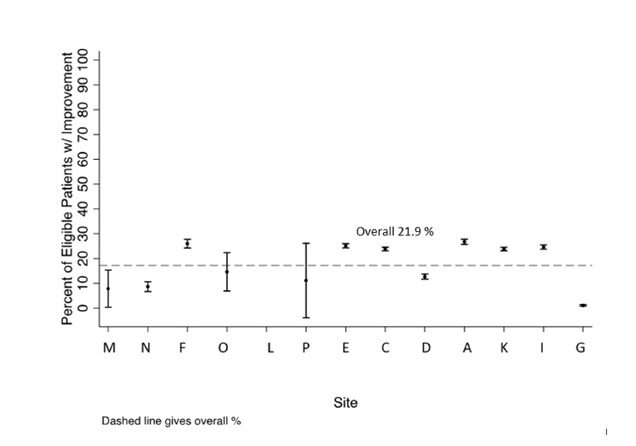


**Figure:**
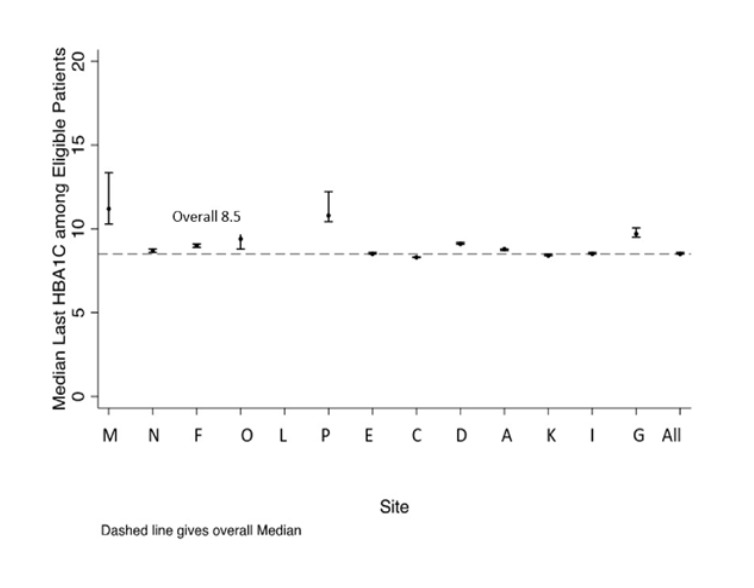


**Figure:**
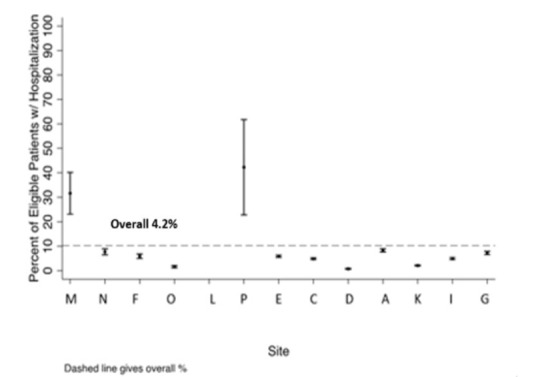


**Figure:**