# Supplementary material for: Testing an Automated Approach to Identify Variation in Outcomes among Children with Type 1 Diabetes across Multiple Sites
Source: Pediatr Qual Saf. 2022 Sep 8;7(5):e602. doi: 10.1097/pq9.0000000000000602 (PMC10997286; doi:10.1097/pq9.0000000000000602)
Supplement: Supplementary file 1 [file pqs-7-e602-s001.docx]

**SDC, Appendix A.** PEDSnet hospitals at the time of the study.

| **Hospital** | **Location** |
| --- | --- |
| Boston Children’s Hospital | Boston, MA |
| Children’s Hospital Colorado | Aurora, CO |
| Children’s Hospital of Philadelphia | Philadelphia, PA |
| Cincinnati Children’s Hospital Medical Center | Cincinnati, OH |
| Nationwide Children’s Hospital | Columbus, OH |
| Nemours Children’s Health System | Wilmington, DE |
| Seattle Children’s Hospital | Seattle, WA |
| St. Louis Children’s Hospital | St. Louis, MO |
