# Supplementary material for: Testing an Automated Approach to Identify Variation in Outcomes among Children with Type 1 Diabetes across Multiple Sites
Source: Pediatr Qual Saf. 2022 Sep 8;7(5):e602. doi: 10.1097/pq9.0000000000000602 (PMC10997286; doi:10.1097/pq9.0000000000000602)
Supplement: Supplementary file 2 [file pqs-7-e602-s002.docx]

**SDC, Appendix B**. Measure Specifications

Years of study: The study will take place over two calendar years –

9/1/2016-831/2017

9/1/2017-831/2018

1. **Denominator**

Has T1D. Number of codes for T1D > T2D and at least one prescription for insulin during the study period. Exclude those with one or more codes for CFRD, steroid induced, gestational diabetes or MODY. Ratio includes anyone with T1D during the study period.

Other inclusion criteria. The be included in the study must have had T1D for 12 months prior to the data collection period and two or more T1D related ambulatory visits in the last year and 0-20 years old (exclude on date of 21^st^ birthday) and ever had prescription for insulin. Denominator needs to be met on the last day of the quarter.

**Outcome measures (numerators)**

1. **2 or more HBA1C results >9%**

Numerator: Count each patient with two or more HBA1C per the year of study of 9.01% or greater in the past year (from last date in the quarter) drawn at the health center of study. Denominator: As defined above and exclude patients with less than two HBA1C results.

1. **Median HBA1C measure**

Numerator is a median of all the patients’ single most recent A1Cs. Denominator: include everyone who meets the denominator definition during the study quarter by the first day of the quarter.

1. **4 or more visits to clinic a year**

Numerator is number of patients with four or more visits with a T1D diagnosis, endocrine, nutrition, social work in the year of study.

1. **Hospitalization for DKA**

Numerator: See body of this publication, methods section

1. **% patients 0-20 years with improvement in HBA1C**

**Denominator:** include everyone who meets the denominator definition during the study quarter by the first day of the quarter and has had two or more hb1cs in the last year. . **Numerator:** If there was a HBA1C in the study period, compare it to the prior HBA1C. If it is 0.5% or more better than the prior one, then there was improvement. If it is not, then there was not improvement. Report the percent with improvement. Use most recent and look back to the one directly before it (limit to outpatient).
