# Supplementary material for: Testing an Automated Approach to Identify Variation in Outcomes among Children with Type 1 Diabetes across Multiple Sites
Source: Pediatr Qual Saf. 2022 Sep 8;7(5):e602. doi: 10.1097/pq9.0000000000000602 (PMC10997286; doi:10.1097/pq9.0000000000000602)
Supplement: Supplementary file 3 [file pqs-7-e602-s003.docx]

**SDC, Appendix C.** Chart Review Data Collection Form

Site: __ Subject ID: __ __ Initials of person entering data: __ __ __

1. Gender:

Male  Female

1. Race *(check all that apply)*:

White/Caucasian  Black/African American  Asian

American Indian/Alaska Native  Native Hawaiian or Pacific Islander

Unknown / not documented  other, specify ________________________

1. Ethnicity: Hispanic/Latino
2. Yes  No  Unknown / not documented
3. Type of Insurance:

Private  Public  Other, specify __________________

1. Was the patient under 21 years of age at any time during Sept.1, 2017 – Aug. 31, 2018?

Yes  No

1. Did they have their 21^st^ birthday in Sept.1, 2017 – Aug. 31, 2018

Yes  No

*If yes, please include data for the remainder of this form only up until the day of his/her 21^st^ birthday. On and after the 21^st^ birthday is not included*

1. Does the patient have a diagnosis of Type 1 diabetes?

Yes  No

***If no****, please explain: _____________ _*

1. How did you know the patient had T1D? Please select one.

Antibodies noted in chart

Documented type in the EMR by a provider (diagnosis, problem list, or provider note)

Other, specify _____________________

1. Did the patient have T1D diagnosis on or before **Sept. 1, 2016**?

Yes  No

**If no:**

- Did the patient have their first T1D diagnosis between **Sept. 1, 2016** – **Aug. 31, 2017**?

Yes  No

***Reminder: If yes,*** *please include only information from 12 months or longer after the T1D diagnosis date. Please do not include any information from fewer than 12 months from the T1D diagnosis date.*

1. Was the patient hospitalized in Sept.1, 2017 – Aug. 31, 2018 for a diabetes related problem

Yes  No

**If yes**

- - - How many times in Sept.1, 2017 – Aug. 31, 2018 was the patient hospitalized for DKA?

Dropdown box: 1-10 which prompts you to enter number in text box

- - - DKA Hospitalization 1: How did you know it was DKA? (select one)

Diagnosis  Lab Data

**If diagnosis of DKA**

- - - - Was there evidence of hyperglycemia (Blood glucose >200 mg/dL (11 mmol/L))?

Yes  No

- - - - Was there evidence of Metabolic acidosis (Venous pH <7.3 or serum bicarbonate <15 mEq/L (15 mmol/L))?

Yes  No

- - - - Was there evidence of Ketosis (Presence of ketones in the blood (>3 mmol/L beta-hydroxybutyrate) or urine ("moderate or large" urine ketones))?

Yes  No

- - - DKA Hospitalization 2: How did you know it was DKA? (select one)

Diagnosis  Lab Data

**If diagnosis of DKA**

- - - - Was there evidence of hyperglycemia (Blood glucose >200 mg/dL (11 mmol/L))?

Yes  No

- - - - Was there evidence of Metabolic acidosis (Venous pH <7.3 or serum bicarbonate <15 mEq/L (15 mmol/L))?

Yes  No

- - - - Was there evidence of Ketosis (Presence of ketones in the blood (>3 mmol/L beta-hydroxybutyrate) or urine ("moderate or large" urine ketones))?

Yes  No

1. Did the patient have two or more T1D related clinic visits in between Sept.1, 2017 – Aug. 31, 2018?

Yes  No

*This includes any visit to endocrine clinic for diabetes related care including routine clinician/provider visit, nurse visit, social work, diabetes education, nutrition, excluding lab visits.*

1. Did the patient have at least 4 T1D related clinic visits in Sept.1, 2017 – Aug. 31, 2018?

Yes  No

*This includes any visit to endocrine clinic for diabetes related care including routine clinician/provider visit, nurse visit, social work, diabetes education, nutrition, excluding lab visits.*

1. Did the patient have two or more results from A1c drawings in Sept.1, 2017 – Aug. 31, 2018?

Yes  No

**If yes:**

- 1. Were there 2 or more drawings that were at least 9.01% or greater in Sept.1, 2017 – Aug. 31, 2018?

Yes  No

1. What was the last A1c result available from Sept.1, 2017 – Aug. 31, 2018?

Text box: _____

1. Did the patient have T1D diagnosis on or before **April 1, 2016**?

Yes  No

1. Was the patient under 21 before **March 31 2018**?

Yes  No

1. Did the patient have 2 or more clinic visits from **April 1, 2017 – March 31, 2018?**

Yes  No

1. Did the patient have an A1c result between **April 1, 2018 – June 30, 2018**?

Yes  No

**If yes:**

- 1. What was the most recent A1c measurement from **April 1, 2018 – June 30, 2018**?

Text box: __________

- 1. What was the most recent A1c measurement from **April 1, 2017 – March 31, 2018**?

Text box: __________  No measurement during this time

- 1. Was this measurement from **April 1, 2017 – March 31, 2018** 9.01% or greater?

Yes  No

- 1. Was there a decrease of 0.5% or more from this measurement to the measurement from April 1, 2018 – June 30, 2018?

Yes  No

***End of chart review section 1, proceed to next section for unblinding/comparison instructions.***

Signature: _______________________________________________ Date: ___/____/_____

*Person completing form dd mmm yyyy*

***Open unblinding envelope/spreadsheet to compare results.***

1. Did the e-measure value for age in Sept.1, 2017 – Aug. 31, 2018 (between 0 and 21 years) match the chart review findings?

Yes  No *If no, explain discrepancy:*

1. Did the e-measure value showing a 21^st^ birthday during Sept.1, 2017 – Aug. 31, 2018 match the chart review findings?

Yes  No  Not applicable *If no, explain discrepancy:*

1. Did the e-measure value for the presence of a T1D diagnosis match the chart review findings?

Yes  No *If no, explain discrepancy:*

1. Did the e-measure value for the date of T1D diagnosis being before Sept. 1, **2016** match the chart review findings?

Yes  No *If no, explain discrepancy:*

1. Did the e-measure value for number of hospitalizations for DKA in Sept.1, 2017 – Aug. 31, 2018 match the chart review finding?

Yes  No *If no, explain discrepancy:*

1. Did the e-measure value for the patient having **2 or more** T1D related clinic visits in Sept.1, 2017 – Aug. 31, 2018 match the chart review findings?

Yes  No *If no, explain discrepancy:*

1. Did the e-measure value for the patient having **at least 4** T1D related clinic visits in Sept.1, 2017 – Aug. 31, 2018 match the chart review findings?

Yes  No *If no, explain discrepancy:*

1. Did the e-measure value for the patient having 2 or more A1cs greater than 9% in Sept.1, 2017 – Aug. 31, 2018 match the chart review findings?

Yes  No *If no, explain discrepancy:*

1. Did the e-measure value for the last A1c result of Sept.1, 2017 – Aug. 31, 2018 match the chart review finding?

Yes  No *If no, explain discrepancy:*

1. Did the e-measure value for improvement by at least 0.5% when **1 A1c is** **greater than 9%** match the chart review finding?

Yes  No *If no, explain discrepancy:*

1. Did the e-measure value for improvement by at least 0.5% when **neither A1cs are** **9% or less** match the chart review finding?

Yes  No *If no, explain discrepancy:*

1. Is there any other pertinent information about the diagnosis, visits, orders, that was observed in the chart review and not already discussed? _________________________________________________

__________________________________________________________________________________

__________________________________________________________________________________

Signature: _______________________________________________ Date: ___/____/____
